# Supplementary material for: Interaction between coxsackievirus B3 infection and α-synuclein in models of Parkinson’s disease
Source: PLoS Pathog. 2021 Oct 25;17(10):e1010018. doi: 10.1371/journal.ppat.1010018 (PMC8568191; doi:10.1371/journal.ppat.1010018)
Supplement: S1 Text — (DOCX) [file ppat.1010018.s001.docx]

# **Supplementary Materials and Methods**

**Transcriptome data and gene expression analysis**

The experimental genome data sets corresponding to α-syn-related, i.e., GSE19496, GSE7621, GSE70368, GSE30792 and GSE116010 were retrieved from the Gene Expression Omnibus database ([http://www.ncbi.nlm.nih.gov/geo/](http://www.ncbi.nlm.nih.gov/geo/query/acc.cgi?acc=http://www.ncbi.nlm.nih.gov/geo/)). Differential expression of genes was analyzed using R (v 3.6.1) (R Foundation for Statistical Computing, Vienna, Austria) and R-studio (v1.2.1335) (RStudio Team 2019). The microarray data were analyzed using the Limma package [1] and the RNAseq data were analyzed using the Deseq2 package [2]. The heatmaps, volcanoplots, and the GO (gene ontology) plot, obtained through data processing, were generated using R as shown in the figure.

**Morphological analysis of microglia**

Morphological analysis of microglia was performed which showed cells colocalizing to VP1 and Iba-1 in the brains of mice on day 7 PI with CVB3 and microglia colocalizing to Iba-1 in control mice. In order to quantify the changes in the complexity, shape, and size of microglia, the fractal dimension [3], lacunarity [4], density [3], span ratio [3] and cell body size [5] were analyzed using the FracLac plugin for ImageJ (NIH, Bethesda, MD). Cell body size and span ratio, which are indexes that change significantly at all locations of the brain, were quantified and displayed in a graph. In addition, microglia process length /cell [6, 7] was measured and analyzed using the AnalyzeSkeleton plugin in ImageJ (NIH, Bethesda, MD) in order to confirm the degree of change in the microglia.

**Evans Blue Dye (EBD) Staining of CVB3 infected mice hearts**

EBD staining was performed as described previously [8]. A day before euthanization, each mouse was injected intraperitoneally with 200 μl of 1% EBD diluted in PBS. Paraffin-embedded heart sections were imaged using the Axioscan Z1 slide scanner (Carl Zeiss, Jena, Germany) at the Three-Dimentional Immune System Imaging Core Facility of Ajou University.

**Hematoxylin and eosin staining of mice tissue sections and cells**

Organs of mice and α-syn OE dSH-SY5Y cells of control and infected with CVB3 were fixed in 4% paraformaldehyde and embedded in paraffin in case of organ of mice. The paraffin blocks were serially sectioned at a thickness of 4 μm. For staining, the deparaffinized sections were stained with hematoxylin (Millipore, Danvers, MA). and eosin (Muto, Tokyo, Japan) solution and exposed to graded ethanol and xylene. After mounting the slides, images of the sections were captured using a slidescanner (Axioscan Z1, Carl Zeiss, Jena, Germany) at the Three-Dimentional Immune System Imaging Core Facility of Ajou University.

# **Supplementary references**

1. Smyth GK. Limma: linear models for microarray data. Bioinformatics and computational biology solutions using R and Bioconductor: Springer; 2005. p. 397-420.

2. Anders S. Differential gene expression analysis based on the negative binomial distribution. Journal of Marine Technology & Environment. 2009;2.

3. Karperien A, Ahammer H, Jelinek H. Quantitating the subtleties of microglial morphology with fractal analysis. Frontiers in cellular neuroscience. 2013;7:3.

4. Karperien AL, Jelinek HF. Fractal, multifractal, and lacunarity analysis of microglia in tissue engineering. Frontiers in bioengineering and biotechnology. 2015;3:51.

5. Torres-Platas SG, Cruceanu C, Chen GG, Turecki G, Mechawar N. Evidence for increased microglial priming and macrophage recruitment in the dorsal anterior cingulate white matter of depressed suicides. Brain, behavior, and immunity. 2014;42:50-9.

6. Morrison HW, Filosa JA. A quantitative spatiotemporal analysis of microglia morphology during ischemic stroke and reperfusion. Journal of neuroinflammation. 2013;10(1):1-20.

7. Morrison H, Young K, Qureshi M, Rowe RK, Lifshitz J. Quantitative microglia analyses reveal diverse morphologic responses in the rat cortex after diffuse brain injury. Scientific reports. 2017;7(1):1-12.

8. Aly M, Wiltshire S, Chahrour G, Osti JL, Vidal S. Complex genetic control of host susceptibility to coxsackievirus B3-induced myocarditis. Genes & Immunity. 2007;8(3):193-204.
