# Supplementary figures and images for: Interaction between coxsackievirus B3 infection and α-synuclein in models of Parkinson’s disease
Source: PLoS Pathog. 2021 Oct 25;17(10):e1010018. doi: 10.1371/journal.ppat.1010018 (PMC8568191; doi:10.1371/journal.ppat.1010018)

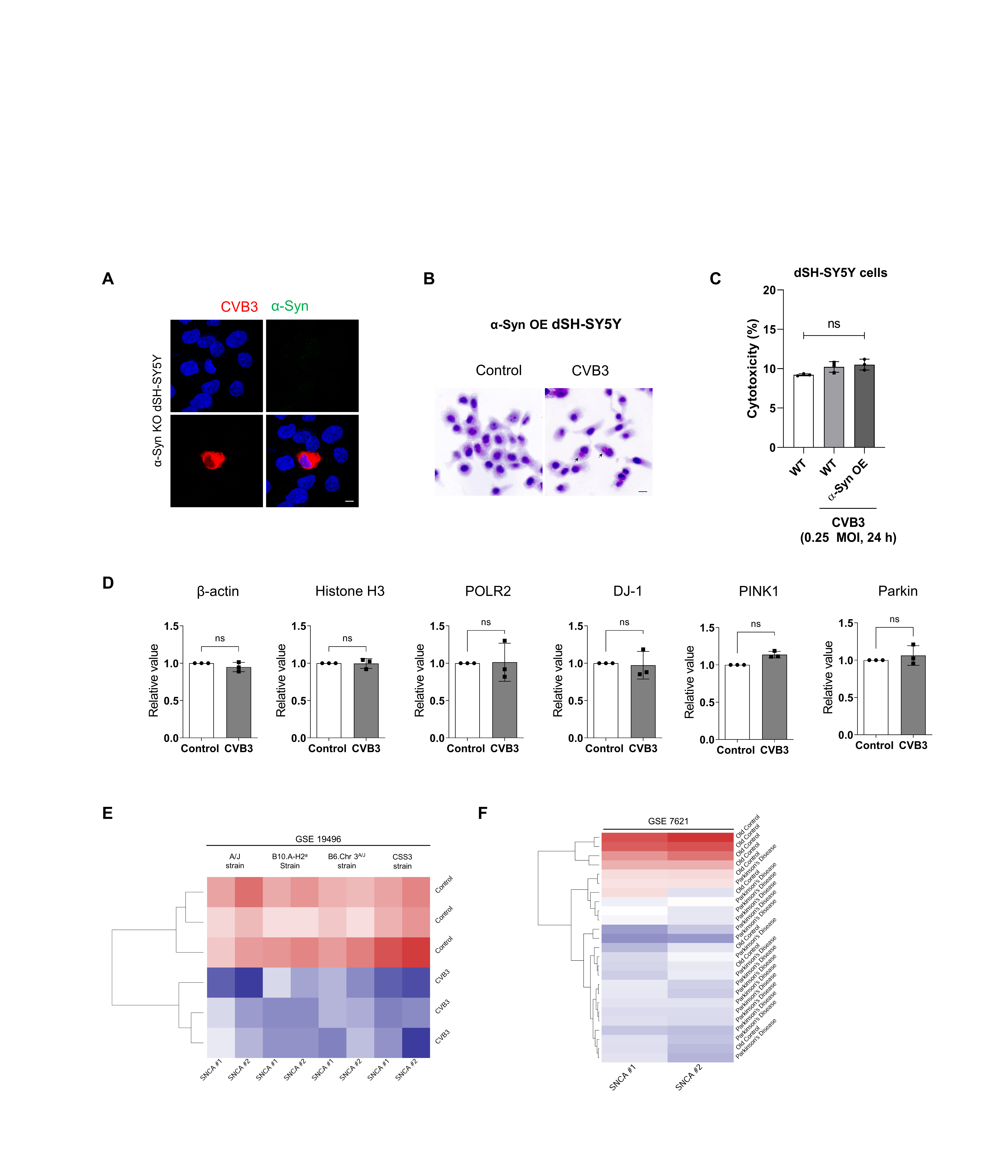

Supplement: S1 Fig — (A) Immunocytochemistry (ICC) images of α-syn KO dSH-SY5Y cells infected with CVB3 (MOI 0.25) (red) for 24 h. Scale bar indicates 10 μm. (B) H&E staining of α-syn OE dSH-SY5Y cells of control and infected with CVB3 (MOI 0.25) for 24 h. Scale bar indicates 20 μm. Arrows indicate eosin positive inclusions of CVB3 infected cells. (C) Cytotoxicity analysis of WT and α-syn OE dSH-SY5Y cells infected with 0.25 MOI of CVB3 for 24 h, by the LDH assay. Values are derived from three independent experiments (n = 3), one-way ANOVA test with Tukey’s multiple comparison test. (D) The relative expression levels of several mRNAs in control and CVB3-infected dSH-SY5Y cells (MOI 0.25) for 24 h [24–26]. Values are derived from three independent experiments (n = 3), unpaired t-test. (E) Gene expression omnibus (GEO) analysis of the relative levels of α-syn in control (n = 3) and CVB3-infected (intraperitoneal (IP) injection of 400 PFUs) mice (n = 3) hearts at day 4 posetinfection (PI) in 4 types of mice strains by array expression profiling (GSE19496). Differential gene expression (DGE) was analyzed using the Limma package in R. The P value of each analysis was as follows: A/J strain (9.20E-07), B10.A-H2a Strain (7.54E-06), B6.Chr 3A/J strain (3.47E-05), CSS3 strain (6.66E-09). (F) The relative levels of α-syn in postmortem brains of normal (n = 9) and patients with PD (n = 16) investigated by array expression profiling (GSE7621). DEG was analyzed using the limma package in R. P value = 0.002 (TIF) [file ppat.1010018.s002.TIF]

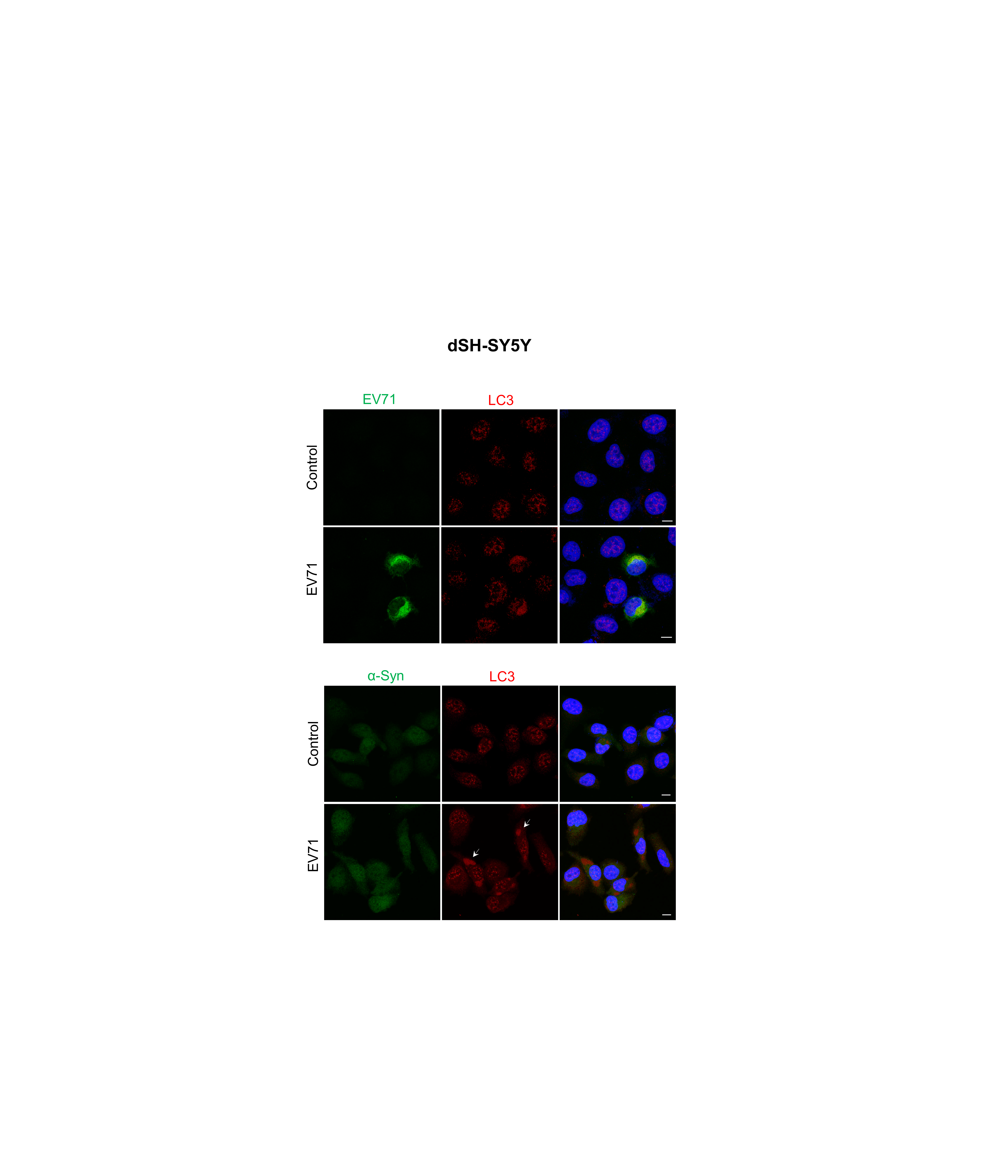

Supplement: S2 Fig — ICC images of WT dSH-SY5Y cells either uninfected (control) or infected with enterovirus 71 (EV71) (MOI 1) for 24 h. Cells were immunostained with indicated antibodies. White arrows indicate LC3-positive aggregates. (TIF) [file ppat.1010018.s003.TIF]

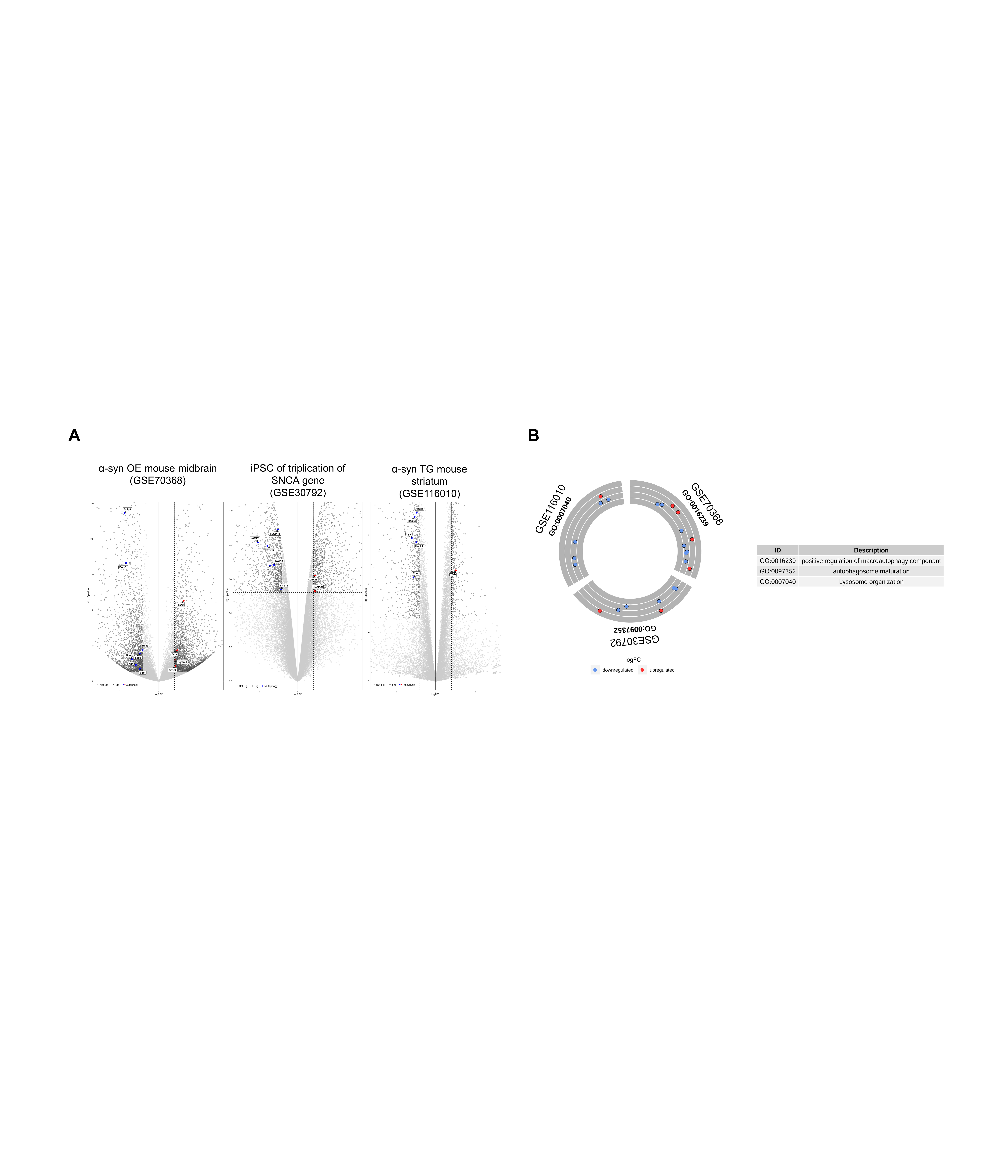

Supplement: S3 Fig — (A) Volcano plots of DEGs between control and α-syn increased environment. (The GEO data sets corresponding to overexpression of human α-syn using a lentiviral vector in mouse midbrain neurons (GSE70368), induced pluripotent stem cells (iPSCs) of α-syn (SNCA) triplicated family (GSE30792) and the mouse striatum tissue of human α-syn transgenic (TG) mice (GSE116010) were analyzed). The light and dark gray dots indicate genes whose expression changed insignificantly and significantly, respectively. The red dots represent up-regulated genes and the blue dots represent down-regulated genes. GSE70368 and GSE116010 were analyzed using the Deseq2 package and GSE30792 was analyzed using the Limma package in R. (B) GO plot for “positive regulation of macroautophagy" (GO:0016239), "autophagosome maturation" (GO:0097352) and "lysosome organization" (GO:0007040) in gene ontology (GO) analysis of GSE70368, GSE30792 and GSE116010 which were higher than the controls in each set. (TIF) [file ppat.1010018.s004.TIF]

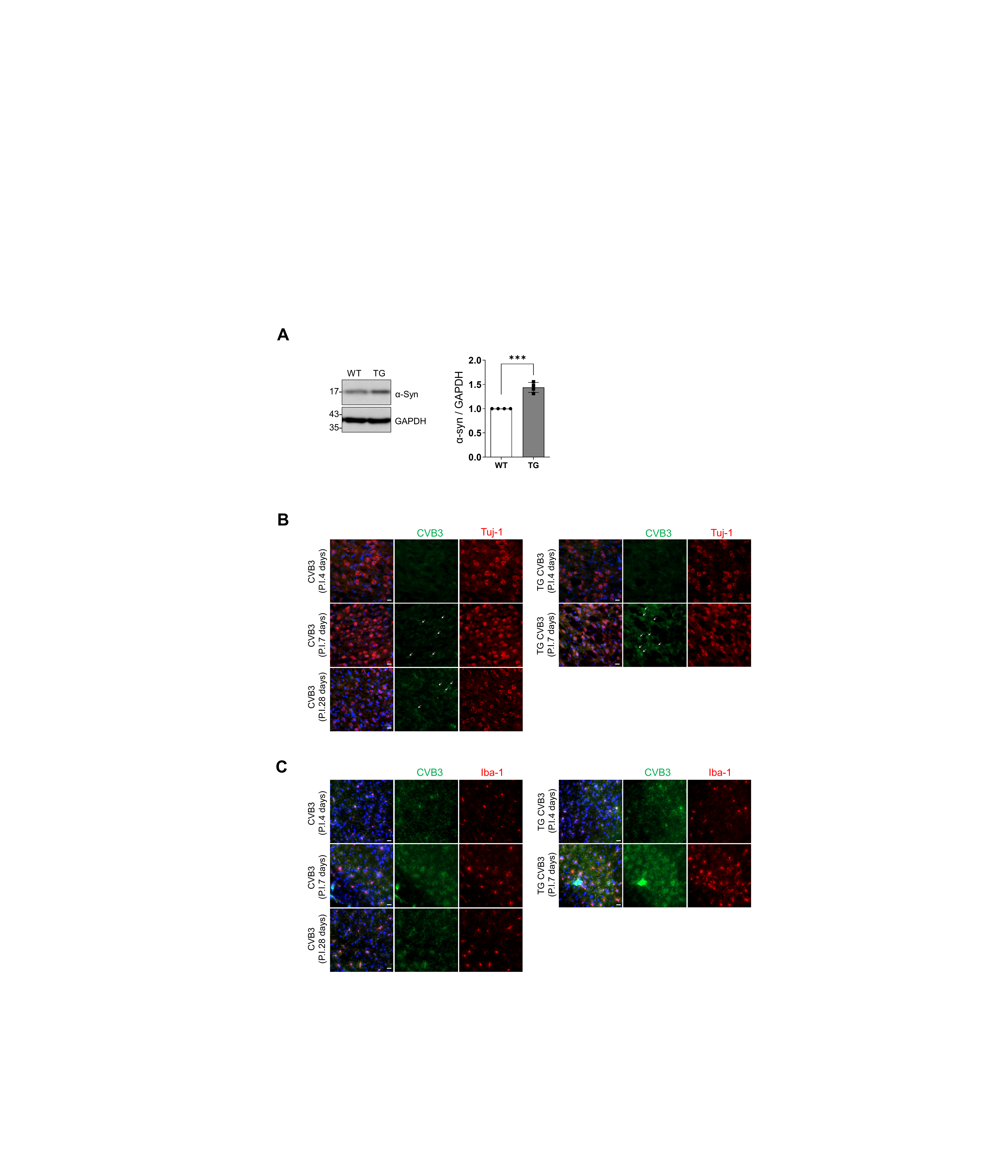

Supplement: S4 Fig — (A) Western blot was performed using control and α-syn TG mice brain lysates. Protein levels were quantified by densitometry. *** P < 0.001, unpaired t-test. IHC images of CVB3-infected mice brains at day 4, 7, and 28 PI (IP injection of 1.0 × 10^6^ PFUs of CVB3). Tuj-1 (thalamus) (B) and Iba-1 (olfactory bulb) (C) positive cells, which were colocalized with VP1 in CVB3 infected WT and α-syn TG mice are shown. White arrows indicate CVB3 infected Tuj-1 positive cells. Blue indicates DAPI. Scale bar indicates 20 μm. (TIF) [file ppat.1010018.s005.TIF]

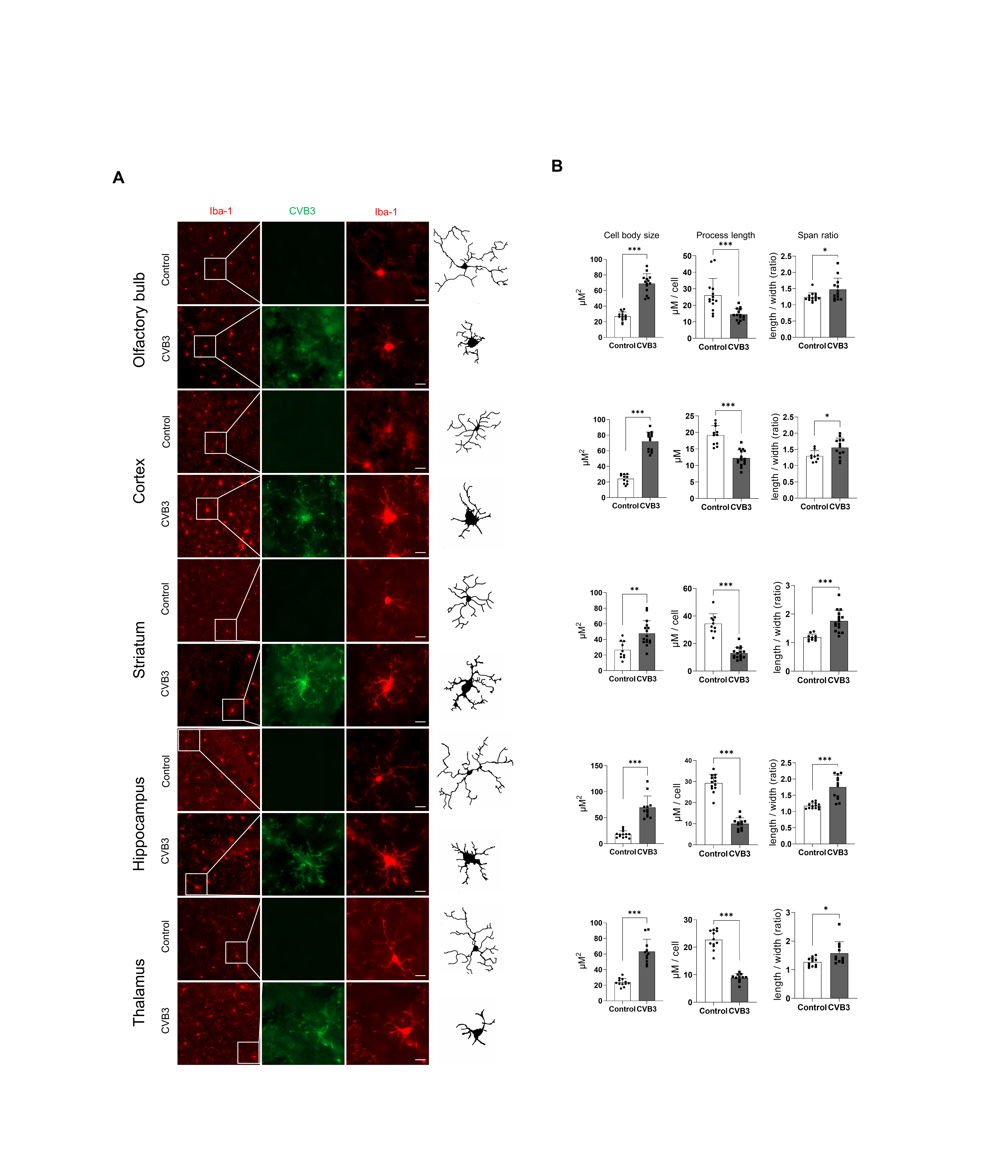

Supplement: S5 Fig — (A) ICC images of control and CVB3-infected mice brains at day 7 PI (IP injection of 1.0 × 10^6^ PFUs of CVB3) and enlarged images with schematic diagram of microglia for analysis (black lined). Scale bar indicates 10 μm. (B) Morphology analysis of VP1-positive microglia in the brains of CVB3-infected mice at day 7 PI. The relative levels of cell body size, process length and span ratio between control and VP1-colocalized microglia. Values are derived from microglia randomly selected from the indicated anatomical structure of three mice. * P < 0.05, *** P < 0.001, unpaired t-test. (TIF) [file ppat.1010018.s006.TIF]

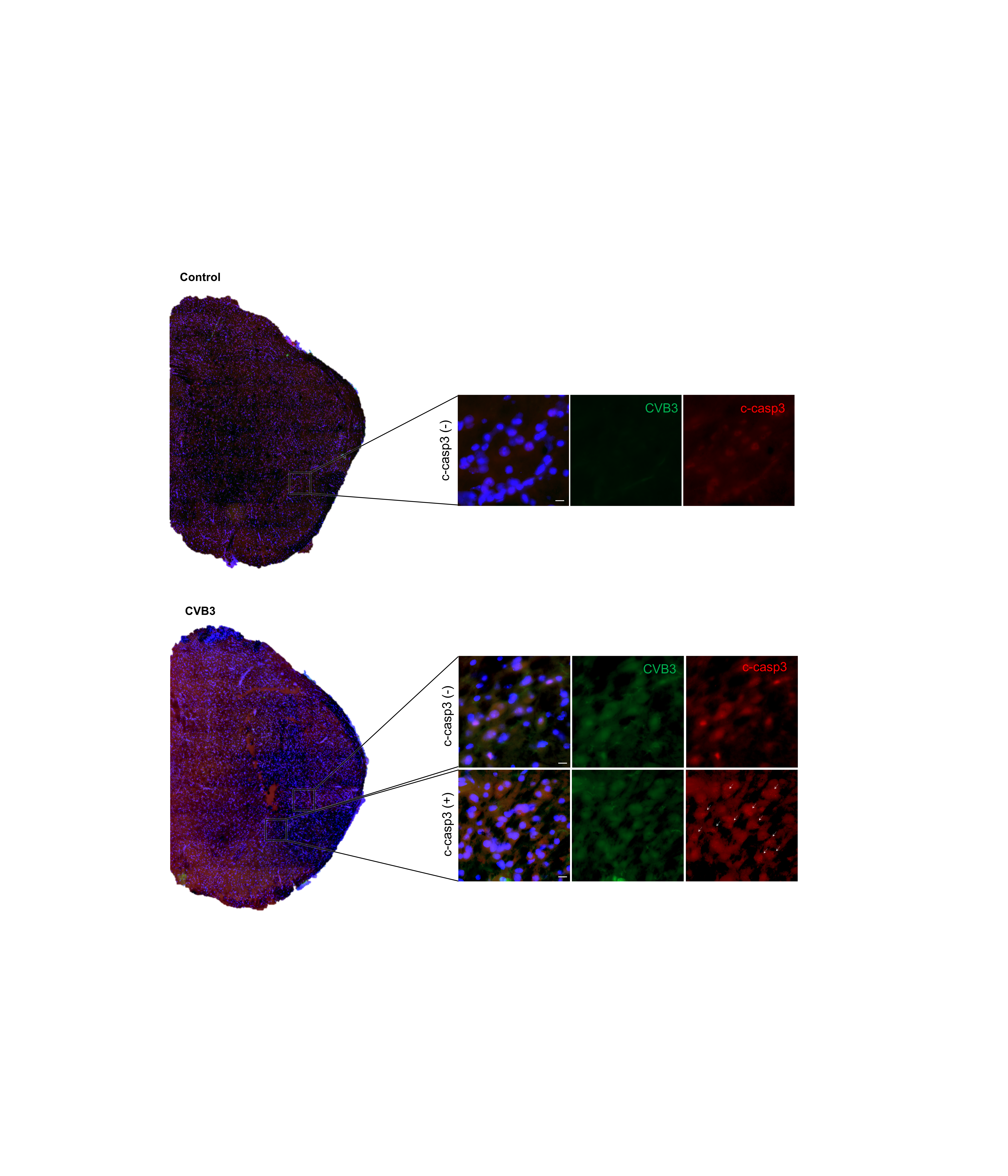

Supplement: S6 Fig — ICC images of control and CVB3-infected mice brains at day 28 PI (IP injection of 1.0 × 10^6^ PFUs of CVB3). White arrows indicate cleaved caspase-3 (c-caspase-3) positive cells. Scale bar indicates 10 μm. Blue indicates DAPI. (TIF) [file ppat.1010018.s007.TIF]

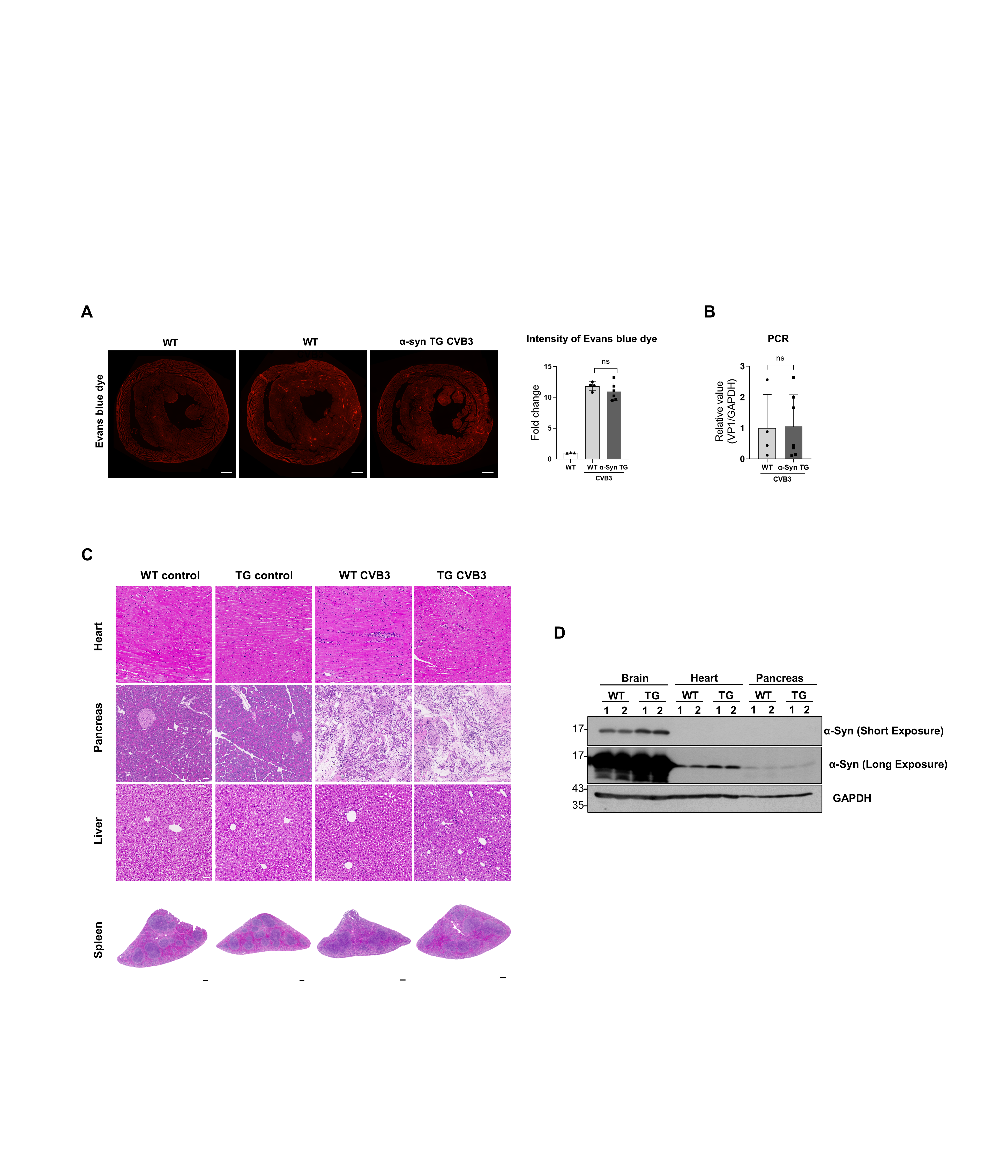

Supplement: S7 Fig — (A) IHC images of Evans blue staining and intensity analysis of control (n = 3), CVB3-infected WT (n = 4), and α-syn TG mice (n = 6) hearts at day 7 PI (IP injection of 1.0 × 10^6^ PFUs of CVB3). Scale bar indicates 500 μm. One-way ANOVA test with Tukey’s multiple comparison test. (B) The relative levels of VP1 between WT (n = 4) and α-syn TG (n = 7) mice hearts at day 7 PI (IP injection of 1.0 × 10^6^ PFUs of CVB3). Unpaired t-test was performed. (C) Hematoxylin and eosin staining of heart, pancreas, liver and spleen in control and CVB3-infected WT, and α-syn TG mice at day 7 PI (IP injection of 1.0 × 10^6^ PFUs of CVB3). (D) Western blot was performed using lysates of brain, heart, and pancreas from control and α-syn TG mice. (TIF) [file ppat.1010018.s008.tif]
