# Supplementary material for: Interaction between coxsackievirus B3 infection and α-synuclein in models of Parkinson’s disease
Source: PLoS Pathog. 2021 Oct 25;17(10):e1010018. doi: 10.1371/journal.ppat.1010018 (PMC8568191; doi:10.1371/journal.ppat.1010018)
Supplement: S1 Table — (DOCX) [file ppat.1010018.s009.docx]

**S1 Table.** Primers for quantitative RT-PCR

| **Gene** | **Forward (5’🡪3’)** | **Reverse (5’🡪3’)** |
| --- | --- | --- |
| VP1 | CACTGGGATTCGTAGATGTT | GTCAGCATGCGTGTACTTTA |
| h_SNCA | AAGAGGGTGTTCTCTATGTAGGC | GCTCCTCCAACATTTGTCACTT |
| m_SNCA | CATGGAGTGACAACAGTGGC | TCCTCACCCTTGCCCATCT |
| h_GAPDH | GAAGGACTCATGACCACAGT | GTAGAGGCAGGGATGATGT |
| m_GAPDH | GTCGGTGTGAACGGATTTGG | ACTGTGCCGTTGAATTTGCC |
| h_ACTB | GGGCATGGGTCAGAAGGATT | AGGTCTCAAACATGATCTGGGT |
| h_H3C1 | ATTCGTCGTTATCAGAAGTCGA | CAGGTTGGTATCTTCGAACAGA |
| h_POLR2 | GCGGAATGGAAGCACGTTAAT | CCCAGCACAAAACACTCCTC |
| h_PARK2 | GTGTTTGTCAGGTTCAACTCCA | GAAAATCACACGCAACTGGTC |
| h_PARK7 | GTAGCCGTGATGTGGTCATTT | CTGTGCGCCCAGATTACCT |
| h_PINK1 | GGAGGAGTATCTGATAGGGCAG | AACCCGGTGCTCTTTGTCAC |

*h* human, *m* mouse, *VP1* viral protein 1, *SNCA* synuclein alpha, *GAPDH* glyceraldehyde 3-phosphate dehydrogenase, *ACTB* actin beta, *H3C1* H3 Clustered Histone, *POLR2* polymerase (RNA) II, *PARK2* parkin, *PARK7* DJ-1, *PINK1* PTEN-induced kinase 1
