# Supplementary material for: Interaction between coxsackievirus B3 infection and α-synuclein in models of Parkinson’s disease
Source: PLoS Pathog. 2021 Oct 25;17(10):e1010018. doi: 10.1371/journal.ppat.1010018 (PMC8568191; doi:10.1371/journal.ppat.1010018)
Supplement: Attachment — Submitted filename: Response to reviewers.docx [file ppat.1010018.s010.docx]

**Responses to Reviewer #1**

**Part I**

1. This reviewer was concerned because there seems to be some blurring with respect to "association" vs. "causation/regulation". For example, it seems inappropriate for the authors to conclude on l. 292: "The present data demonstrate that CVB3 interacts with alpha-synuclein to promote PD."

**[Response]** As suggested by reviewer #1, we have modified the sentence to “CVB3 infection and α-syn expression have a mutual effect on each other, which may promote PD”. An explanation of this sentence is provided in the discussion section.

2. Also, one wonders how different the results are compared to the effects of other picornaviruses.

**[Response]** As suggested by reviewer #1, we agree that further studies are required to confirm whether our observations are specific to CVB3 or not. In order to confirm whether the observations are specific to CVB3, we used poly IC, an artificial analog to mimic RNA viral infection. Furthermore, we have performed experiments using another picornavirus, enterovirus71 (EV71). Although EV71 infection induced LC3 positive inclusions like CVB3 infection, these inclusions were smaller than those in CVB3 infected cells and did not colocalize with α-syn, suggesting that our observations are CVB3-specific. We have included these data in the results section (p9, line 152-155) and supplementary figure S2.

3. An issue that this reviewer has with the manuscript is what looks like incomplete details regarding some the methods. For example:

a) l. 110 - ① it is unclear what the timing was when there was a decrease in alpha-synuclein mRNA, and ② whether other mRNAs were examined. ③ Could this just be related to destruction of the cells?

**[Response]** ① We performed real time RT-PCR 24 h after viral infection. We have added this information in the legend of figure 1. ② Additionally, we performed real time RT-PCR to examine the expression of other mRNAs. We did not observe any change in the expression of some genes such as β-actin, histone H3, polr2, DJ-1, PINK1, and parkin, suggesting that a decrease in α-syn mRNA might not be due to host shutoff phenomenon. We have included this information in supplementary figure S1B. ③ We performed the LDH assay 24 h after viral infection and we did not observe any significant cytotoxicity, thereby concluding that it was not due to cytotoxicity. We have included this result in supplementary figure S1A. Nevertheless, we cannot exclude these possibilities completely. Accordingly, we have discussed regarding them in the discussion section (p18, line 355-357).

b) l. 116 - alpha-synuclein protein increased in infected cells and decreased in neighboring cells. Isn't there translational shutoff of picornaviruses during infection? The ICC images may not be the best way to see this.

**[Response]** As suggested by reviewer #1, host shutoff phenomenon may occur. Accordingly, we examined the expression of several mRNAs by RT-PCR. The mRNA levels were not changed upon CVB3 infection. Currently, as we cannot exclude the effect of host shutoff phenomenon induced by CVB3 completely, we have discussed the possibility of this event in the discussion section (p18, line 355-357).

c) The authors use overexpressing alpha-synuclein cells and mice. A previous study by Masliah et al found that alpha-synuclein transgenic mice have neurodegeneration. Do the cells and mice used in this investigation also have abnormalities, and could these influence their results? In Fig. 6K, It would be valuable to see the survival rate or pathology noted for the uninfected alpha-synuclein transgenic mice.

**[Response]** As commented by reviewer #1, since the time Masliah et al. developed transgenic mouse lines expressing WT α-syn under the control of PDGF promoter [1], many transgenic mouse lines expressing WT α-syn under the control of various promoters such as prp and thy-1 [2-4] have been developed. Although Masliah et al. reported that these transgenic mice had intraneuronal inclusions immunoreactive with antibodies to human α-syn at 2 months of age, no histological abnormalities including neurodegeneration were observed at that age in many other transgenic lines expressing WT α-syn [2-4]. In the transgenic mouse line we used in this study, the expression of WT α-syn was driven by the NSE promoter and the expression of human α-syn was not as high as that in other TG lines (Fig S4A). Although it has not been characterized in detail, no clinical phenotype, including weight loss, was observed during our experimental time points (1 months), suggesting no obvious toxicity related to the overexpression of α-syn. We have mentioned it in the results section (p12, line 240-p13, line 242). Additionally, we have included information regarding survival rate and weight change in the absence of CVB3 infection in figure 6K. In the case of α-syn OE cell line, we did not observe any abnormalities (compared with the control line). The EM findings also support this conclusion.

d) The authors should provide virus titers in order to examine CVB3 replication.

**[Response]** As suggested by reviewer #1, we have measured the viral titers using the plaque assay and added these data in figures 4A and 6A.

e) l. 261 - Did the authors examine other tissues besides the heart and brain in the CVB3-infected mice?

**[Response]** As suggested by reviewer #1, we examined other tissues, including heart, liver, pancreas and spleen. Although CVB3-infected mice showed inflammation of some organs, we did not observe any differences in histological abnormalities between WT and α-syn TG mice. We have included these data in supplementary figure 7 and in the results section (p14, line 283-285).

f) Fig. 4H - At what time were the cells examined?

**[Response]** We have already mentioned this information in the legend of figure 4. “(H) Cytotoxicity analysis using lactate dehydrogenase assay of WT and α-syn OE dSH-SY5Y cells infected with 0.25 MOI CVB3 for 30 h.”

**Part II**

Titers of infections virus are necessary.

**[Response]** See the response to the reviewer#1 comment 3d.

It would be valuable to carry out experiments in alpha-synuclein knock-out cells and mice.

**[Response]** We agree with the suggestion of reviewer #1. However, it is difficult for us to obtain α-syn KO mice and study it within the limited time. Accordingly, we would prefer to conduct this investigation in a separate future study. Instead, we have generated α-syn KO cell line using CRISPR/Cas9 system. Although we could not perform all the experiments using this cell line, we have performed two main experiments. In CVB3-infected α-syn KO cells, α-syn fibril-like structures were not observed in EM analysis. In addition, the maturation of LB-like structures was delayed. We have included these data in figures 2G and 3. Nevertheless, we intend to conduct further investigation using this cell line and with α-syn KO mice in a separate future study.

It would be important to note the survival and pathology of alpha-synuclein transgenic mice.

**[Response]** See the response to the reviewer #1’s comment 3c.

Timing of data shown needs to be included.

**[Response]** We have added this information in the figure legends.

Controls for some experiments are not shown. For example, the examination of other mRNAs or proteins; a comparison of CV3B with other picornaviruses.

**[Response]** See the response to the reviewer#1 comment 2 and 3a-b.

**Responses to Reviewer #2**

**Part I**

1. Is it possible that in the cell cultures at least, the loss of cells due to the CVB3 infection skews the a-syn expression level of the whole culture downward?

**[Response]** We performed LDH assay 24 h after viral infection and we did not observe any significant cytotoxicity, thereby concluding that it was not due to cytotoxicity. We have added this data in supplementary figure S1A. Nevertheless, we cannot exclude the possibility of cytotoxicity completely. Accordingly, we have discussed the possibility in the discussion section (p18, line 355-357).

2. As a-syn overexpression itself has been shown to impair autophagic flux [Sarkar et al. (2021) PLOS Genetics 17(2):e1009359], it seems likely that the two processes affecting autophagy may result in an environment favoring generation of the necessary replication organelles for CVB3 replication and for generation of inclusion bodies.

**[Response]** We thank reviewer #2 for this suggestion, and it seems likely that the two processes affect autophagy. Accordingly, we have discussed this possibility in the discussion section (p20, line 417-419) and cited the relevant reference.

3. Have the authors considered that evidence in human cardiomyopathy and in human type I diabetes combined with mouse models of these diseases suggests that enteroviruses may persist at extremely low levels but still cause pathology (Kim et al. J Virol. 2005 Jun;79(11):7024-41; Bouin et al. Circulation. 2019 May 14;139(20):2326-2338; Oikarinen et al. Viruses. 2020 Jul 11;12(7):747; Krogvold et al. Diabetes. 2015;64:1682–1687? It is extremely hard to detect the enterovirus by immunohistochemistry in these studies. In the brain in particular, the viral load is very low in adult mice, although infection of neonatal mice has been shown to induce persistence of the virus in the brain for 3 months post inoculation (Feuer et al J Virol. 2009;83(18):9356-9369.) In these studies, the use of RT-PCR allowed detection of the viral genome in infected tissue but only very sensitive detection of capsid protein demonstrated the presence of viral protein. The immunohistochemical method of detection of virus infection is inherently less sensitive than a method which allows amplification of the viral genome. This is not to say that the authors’ hypothesis is without merit, just that it cannot rule out the presence of the virus in the region where pathology occurs without doing an assay involving amplification of the low viral signal.

The observation that a-syn overexpression and CVB3 infection could result in Lewy body type inclusions in neuronal cells and that a-syn overexpression increased CVB3 replication does suggest the effects of both factors upon the induction of autophagy and the late stage autophagy block by CVB3 preventing clearance of aggregates may generate the type of pathology seen in PD. It is less certain that CVB3 and other enteroviruses may act as a trigger, rather than a persistent inducer of the inclusions as the authors have not done the type of assay noted for detecting the very low level chronic infection seen in other diseases such as cardiomyopathy and type 1 diabetes, specifically RT-PCR. As the authors have generated sufficient data to make this rather difficult process worthwhile in this animal model, the study merits publication but should discuss the limitations of immunohistochemistry in detection of the unusual persistence demonstrated with the coxsackievirus B viruses.

**[Response]** We thank reviewer #2 for this suggestion. We have discussed this possibility in the discussion section and cited the relevant reference (p19, line 384-388).

**Responses to Reviewer #3**

**Part I**

1. The authors make a number of claims based on morphology but since the whole paper suffers from extremely poor image quality this is really hard to be confirmed by looking at those figures, even with a looking glass. In most cases microscopic images are less than 1 cm on a letter page meaning that in the final paper they will be even smaller.

I propose that the authors present a novel manuscript with high-resolution images that will allow to better evaluate the claims they are putting forward.

**[Response]** As suggested by reviewer #3, we have tried our best to enlarge the images in the figures to show larger high-resolution images.

**Part II**

In many cases, appropriate controls are missing. For example,

1. it would be important to show differences in a-syn aggregates also in uninfected overexpressing SHSY cells (Fig 1).

**[Response]** After differentiation induced by retinoic acid, α-syn OE SH-SY5Y cells exhibit a few number of small α-syn aggregates, which is similar to the findings observed in A53T α-syn OE SH-SY5Y cells in our previous study [5]. Nevertheless, it is noteworthy that α-syn aggregates in infected cells (large inclusion body-like structure) are different from those in uninfected cells. To avoid confusion in comparison with Fig 1G, we replaced them (Figs 1G and K) with new ones.

2. The authors repeatedly claim lewy body (LB)-like structures – they should not because they do not present appropriate evidence. For example PMID 32339655 shows should look in EM. Their TEM pictures in Fig. 2 are not revealing in the absence of immunogold-labeling for a-syn and CXB3 - if they could convincingly show fibrillar a-syn structures immunolabeled in TEM it would increase the paper’s impact.

**[Response]** While immune-EM is a powerful tool for detecting the sub-cellular localization of a protein, it is technically challenging, and requires rigorous optimization of tissue fixation and processing methods. We have tried our best to optimize tissue processing for several months before and after submission, however, we have failed at it. Although we will continue to optimize this processing, it might be difficult for us to show it in this study within the limited time. Instead, we have generated an α-syn KO cell line using the CRISPR/Cas9 system. In CVB3-infected α-syn KO cells, α-syn fibril-like structures were barely observed in EM analysis, supporting our results indirectly. We have included the data in figure 2G.

3. The animal studies are important and the finding that the tgSyn animals exhibit a higher mortality (I assume a normal life expectancy of this strain which is not reported) is interesting,

**[Response]** The transgenic mouse line we used in this study was developed under the control of NSE promoter and the expression of human α-syn was not as high as that in other TG lines (Fig. S4A). Although it has not been characterized in detail, no clinical phenotype, including weight loss, was observed during our experimental period (1 months), suggesting no obvious toxicity related to the overexpression of α-syn. We have mentioned this information in the results section. Additionally, we have included the data regarding survival rate and weight change in the absence of CVB3 infection (Fig. 6K).

4. As I said the asyn stainings claimed to be different in CXB3 infected vs. control suffer from poor quality and, in my opinion, are at this point inconclusive and not supporting the authors’ claims.

**[Response]** See the response for the reviewer #3’s comment Part II #1.

There are many typos or grammatical mistakes.

**[Response]** Although we have edited our manuscript with the help of a native speaker, we have re-modified our manuscript with the help of another native speaker to polish the text again.

In the methods section I miss how the decrease of TH-positive neurons upon CXB3 infection (Fig. 6I) was quantified (stereology?).

**[Response]** We have included the detailed methodology to quantify TH positive neurons in the materials and methods section (p29, line 618-622).

**References**

1.	Masliah E, Rockenstein E, Veinbergs I, Mallory M, Hashimoto M, Takeda A, et al. Dopaminergic loss and inclusion body formation in alpha-synuclein mice: implications for neurodegenerative disorders. Science (New York, NY). 2000;287(5456):1265-9. Epub 2000/02/26. doi: 10.1126/science.287.5456.1265. PubMed PMID: 10678833.

2.	Lee MK, Stirling W, Xu Y, Xu X, Qui D, Mandir AS, et al. Human alpha-synuclein-harboring familial Parkinson's disease-linked Ala-53 --> Thr mutation causes neurodegenerative disease with alpha-synuclein aggregation in transgenic mice. Proceedings of the National Academy of Sciences of the United States of America. 2002;99(13):8968-73. Epub 2002/06/27. doi: 10.1073/pnas.132197599. PubMed PMID: 12084935; PubMed Central PMCID: PMCPMC124407.

3.	Giasson BI, Duda JE, Quinn SM, Zhang B, Trojanowski JQ, Lee VM. Neuronal alpha-synucleinopathy with severe movement disorder in mice expressing A53T human alpha-synuclein. Neuron. 2002;34(4):521-33. Epub 2002/06/14. doi: 10.1016/s0896-6273(02)00682-7. PubMed PMID: 12062037.

4.	Chandra S, Gallardo G, Fernandez-Chacon R, Schluter OM, Sudhof TC. Alpha-synuclein cooperates with CSPalpha in preventing neurodegeneration. Cell. 2005;123(3):383-96. Epub 2005/11/05. doi: 10.1016/j.cell.2005.09.028. PubMed PMID: 16269331.

5.	Choi YR, Kim JB, Kang SJ, Noh HR, Jou I, Joe EH, et al. The dual role of c-src in cell-to-cell transmission of alpha-synuclein. EMBO Rep. 2020;21(7):e48950. Epub 2020/05/07. doi: 10.15252/embr.201948950. PubMed PMID: 32372484.
