# Supplementary material for: Interaction between coxsackievirus B3 infection and α-synuclein in models of Parkinson’s disease
Source: PLoS Pathog. 2021 Oct 25;17(10):e1010018. doi: 10.1371/journal.ppat.1010018 (PMC8568191; doi:10.1371/journal.ppat.1010018)
Supplement: Attachment — Submitted filename: Responses to reviewers.docx [file ppat.1010018.s011.docx]

**Response to Reviewer #1**

**Part I**

1. ① The authors note that alpha-syn levels were decreased in the CVB3-infected mouse heart and brain of PD patients; one wonders whether this is the case because there is a change in cell types as well as cell death in the heart and brain. ② CVB3-infected cells growing in vitro had increased levels of alpha-syn compared to the bordering cells - is this because of interferon produced by the infected cells? ③ Was the alpha-syn really increased OR was it just aggregated?

**[Response]** ① Since we analyzed open source database to support our experimental results, we could not answer it completely. However, it may be speculated that the similar mechanisms seem to work regardless of cell types. ② We could not also answer the reason why CVB3 differently affected α-syn expression between infected cells and uninfected neighboring cells differently. We thought that cytokines or chemokines such as interferons released by CVB3 infected cells may affect α-syn expression in the neighboring cells. In preliminary study, we observed that several chemokines such as CXCL10 and CXCL11 were increased by CVB3 infection. When we treated CVB3-infected dSH-SY5Y cells with CXCR3 inhibitor (AMG487), which the binding of CXCL10 and CXCL11 to CXCR3, we did not observe any difference of α-syn expression with or without CCR4 inhibitor (Figure 1 for reviewers only), suggesting that CXCR3 may not be responsible. We thought that this observation was very important. However, this study was more focused on the role of CVB3 in α-syn aggregation. Accordingly, we would prefer to conduct this investigation in a separate future study. ③ Given that the intensity of α-syn in the infected cells was increased, α-syn was increased and further aggregated.

Figure 1 for reviewer only

2. This reviewer's impression is that LBs are eosinophilic inclusion bodies. Is this the case with CVB3 infection? If so, it would be best for the authors to note this.

**[Response]** As suggested by the reviewer #1, we performed H&E staining after CVB3 infection. We observed that eosionophilic inclusion in cells presumed to be infected with CVB3 (Eosinophlic inclusions filled the cytoplasm and pushed the nucleus aside, creating a half-moon appearance). We added it in Fig S1B.

3. There was a statistical difference in the titer (l. 210) at 30 hours. One wonders what titer differences would be present at other time points. What would be helpful is if the authors put some of the P values within the body of the manuscript.

**[Response]** Until 24 h, no cytotoxicity difference between mock and α-syn OE SH-SY5Y cells was observed. However, when both cells were infected with CVB3 for more than 30 h, the cytotoxicity of CVB3 infection was more severe in α-syn OE SH-SY5Y cells. We have already described the P values if necessary.

4. The authors note that survival of the alpha-syn tgnic mice "was poorer" than that of WT mice. I'm not sure what the titer difference was since I could not see the figure well; however, this study should compare the transgenic mice with littermates that are not transgenic. The Methods section failed to mark it unclear whether this was always carried out.

**[Response]** We have compared the transgenic mice with wild-type (WT) littermates or WT C57BL/6N mice as controls. We have already described it in the Materials and Methods section (L.470-471).

5. L. 285: "These results suggested..." It would be valuable if the data made the results clear.

**[Response]** “These results suggested that CVB3 infection in the mouse brain caused neuronal death, including loss of dopaminergic neurons located in the substantia nigra (Fig. 6G-H and 6I-J), and that α-syn accelerated the replication of CVB3 (Fig. 6A-B and 6D-E) and CVB3-induced neuronal death (Fig. 6F-G).”. We have already shown the data in Fig 6.

6. l. 292: Why do the authors think that there was no colocalization of alpha-syn and CVB3 VP1 at day 7. Shouldn't the virus cause an early infection? I was unable to see Fig. 7C well.

**[Response]** Currently, we could not answer this question completely. Instead, we have already described the possibility in the discussion section (L.370-390).

7. L. 317: I don't think the authors have shown that CVB3 "promotes" PD. l. 444: "our findings clarify the mechanism of LB formation and the pathogenesis of PD associated with CVB3 infection." This reviewer would like to see more data. For example, it would be valuable if there was evidence of low levels of CVB3 genome by PCR in PD brains.

**[Response]** We have already cited the reference showing virus-like particles and coxsackievirus antigen in the brainstem neurons of PD in the introduction section [1] (L. 92-93). Nevertheless, we deleted the sentence “which promote PD” to avoid confusion.

**Part II**

The figures need to be easily readable.

**[Response]** We have tried our best to provide high-resolution images. Published images may be higher-resolution images.

The titers should be carried out at more than one time point.

**[Response]** See the response to the reviewer#1 comment 3.

**Response to Reviewer #2**

C57BL/6 mice are not noted for long term CVB3 infections but are known to have high levels of acute pathology both in heart and pancreas. The clear evidence of pancreatic disease (both in pathology and body weight) induced by the high titer i.p. inoculum likely plays a part in the increased mortality. Is the more rapid mortality in the transgenic mice due to the increased a-syn expression in the brain alone? As a-syn has been shown to be expressed in islet beta cells (Steneberg, et al. 2015, Diabetes 62:2004–2014), it is possible that expression of a-syn at higher levels than normal in these beta cells could lead to increased susceptibility to CVB3 infection. Alteration of beta cell function could explain the increased mortality.

**[Response]** We used α-syn transgenic (TG) mice overexpressing human α-syn under the control of the neuron specific enolase (NSE) promoter. NSE is expressed in most terminally differentiated neurons and neuroendocrine cells, but not in other cell types. Many studies have used this promoter to express target genes in neurons specifically [2, 3]. Although α-syn is endogenously expressed in islet beta cells, transgenic α-syn may not be expressed in islet beta cells. We also confirmed that endogenous α-syn expression in the pancreas was much lower than that in the brain and there was no difference of endogenous α-syn expression in the pancreas from both WT and TG mice (Figure 2 for reviewers only). Accordingly, the more rapid mortality in the transgenic mice may not be due to the increased α-syn expression in the pancreas.

Figure 2 for reviewers only

**Part III**

I suggest the authors provide a sentence or two discussing the role of pancreatic disease in the morbidity and mortality of the CVB3 infected mice (demonstrated by the pancreatic pathology and the body weight loss) and the possibility that a-syn expression in the pancreas leading to a significant increase in CVB3 replication in those tissues explains the higher morbidity and mortality in the a-syn OE transgenic mice. In addition, the crystalline arrays in the dSH-SY5Y cells should be in discussions of Figure 2E in the text.

**[Response]** As suggested by the reviewer #2, we added the explanation of the pancreatic tissue damage of CVB3 infected mice (L. 282-285). Given that transgenic α-syn was not expressed in the pancreas, we did not add the possibility suggested by the reviewer #2. Additionally, we also added the explanation of viral particles shown in Fig 2E (L. 162-163).

**Response to Reviewer #3**

**Part II**

Figure 1. ① why do the authors measure “relative intensity of a-syn“ rather than counting aggregates? ② The representative images 1G/K do not look convincingly different infected vs. non-infected. ③ For ICC, the authors need to show a control that the anti-syn antibody does not crossreact with CVB3.

**[Response]** ① As we have already described in the former sentence, we would like to explore whether CVB3 affects α-syn in various parts. Accordingly, first, we explored whether CVB3 affects α-syn expression or whether CVB3 affects α-syn aggregation. Since CVB3 VP1 colocalized with α-syn aggregation almost completely, counting aggregates just meant the number of cells infected by CVB3. ② Although the formation of α-syn aggregates looks less clear in dSH-SY5Y cells (upper figures), the formation of α-syn aggregates looks clear in dSH-SY5Y cells overexpressing α-syn (lower figures). In figure 1K, we did not infect dSH-SY5Y cells with CVB3. ③ As suggested by the reviewer #3, we performed ICC using α-syn KO dSH-SY5Y cells and we did not observe the crossreactivity of anti-α-syn antibody with CVB3. We added it in the result section (L.110-111) and Fig S1A.

Figure 2. The authors state that they were not able to stain the fibril-like structures with a-syn immunogold antibodies. Contrary to the authors, I interpret the failure to see TEM immunogold labeling for a-syn as evidence that the fibril structures are NOT consisting of a-syn which makes a central claim of their paper fall apart. Furthermore the term LB-like is not suited for spherical structures such as the ones pointed to by arrows in Fig 2G/H since LBs have a concentric structure. Without having certainty about the identity of the observed fibril-like structures, the significance of the reported findings drops. The a-syn KO line results are only indirect and insufficient. The structures pointed at with the arrows in Figure 2 could thus be anything. In the absence of such a conclusive evidence, the authors should not continue to claim in several parts of the paper, including the abstract, that CVB3 causes a-syn aggregates. The sentences in the abstract “This study elucidated the mechanism of Lewy body formation and the pathogenesis of PD associated with CVB3 infection.“ or in the autor summary “Our findings clarify the mechanism of LB formation...“ are therefore wrong.

**[Response]** Although we have tried our best to optimize the procedure for immune-EM several times, we failed to optimize fixation protocols. So, we could not preserve the cellular morphology after immunostaining, which does not mean the failure to see TEM immunogold labeling for α-syn as the reviewer #3 thought. In a previous study, Mahul-Mellier et al. performed confocal and correlative light EM (CLEM) imaging analysis to characterize LB-like inclusions. Although LB-like inclusions show round structures by ICC against pS129-α-syn, they contained fibrillar α-syn with mitochondria and several type of vesicles by EM (figure 2 in [4] ) and fibrillar α-syn was confirmed by immunoEM (figure 1K in [4] ), which were very similar with our findings. Accordingly, the structures pointed at with the arrows in Figure 2 may not be anything. We have already described their findings and cited this reference in the discussion section (L. 333-336). In addition, we have also described the limitation of our results (L. 344).

Figure 3A – the pS129 staining is too weak and does not show aggregates – the attempt of “staging“ therefore seems premature.

**[Response]** “We classified them into four stages based on the staining pattern of LC3” (L. 181-182). We classified them into four stages based on the staining pattern of LC3, not of pSer129 α-syn. Additionally, we used dSH-SY5Y cells, which express small amount of endogenous α-syn to rule out the effect of α-syn overexpression. Therefore, the pS129 staining seems to be weak. In α-syn OE SH-SY5Y cells, α-syn aggregates was clearly observed (Fig. 2B).

Fig 4A. the difference in virus titer measured by plaque assay is not even one order of magnitude, but only minimal. This could be thus entirely be due to a selection artifact when generating the stable overexpressing cell line. The plaque assay from Fig 6A is more convincing (two orders of magnitude titer difference) – but how do the authors then explain this discrepancy? As also mentioned by another reviewer, titer measurements in a-syn ko cells or mice should be performed to corroborate the claim of an interaction of a-syn with CVB3 replication

**[Response]** As suggested by the reviewer #3, we re-performed plaque assay adding α-syn KO cells. Nevertheless, we could not observe much higher difference between WT and α-syn OE cells in plaque assay, which is similar with our previous finding. Instead, we observed less replication of CVB3 in α-syn KO cells, compared with WT cells. We added it in Fig 4A.

Figure 7: the staining of CVB3 and a-syn is too weak to support the authors’ claim that CVB3 infection supports a-syn aggregation in vivo, let alone LB formation.

**[Response]** To avoid confusion, we replaced the term “α-syn inclusions” to “a few neurons containing α-syn **accumulations** in the cell body”. In addition, we replaced the term “α-syn inclusions” to “α-syn accumulations in the cell body” in all of the description of *in vivo* data.

**References**

1.	Dourmashkin RR, McCall SA, Dourmashkin N, Hannah MJ. Virus-like particles and enterovirus antigen found in the brainstem neurons of Parkinson’s disease. F1000Research. 2018;7. doi: 10.12688/f1000research.13626.1.

2.	Forss-Petter S, Danielson PE, Catsicas S, Battenberg E, Price J, Nerenberg M, et al. Transgenic mice expressing beta-galactosidase in mature neurons under neuron-specific enolase promoter control. Neuron. 1990;5(2):187-97. Epub 1990/08/01. doi: 10.1016/0896-6273(90)90308-3. PubMed PMID: 2116814.

3.	Pohlkamp T, Steller L, May P, Gunther T, Schule R, Frotscher M, et al. Generation and characterization of an Nse-CreERT2 transgenic line suitable for inducible gene manipulation in cerebellar granule cells. PloS one. 2014;9(6):e100384. Epub 2014/06/21. doi: 10.1371/journal.pone.0100384. PubMed PMID: 24950299; PubMed Central PMCID: PMCPMC4065071.

4.	Mahul-Mellier AL, Burtscher J, Maharjan N, Weerens L, Croisier M, Kuttler F, et al. The process of Lewy body formation, rather than simply alpha-synuclein fibrillization, is one of the major drivers of neurodegeneration. Proceedings of the National Academy of Sciences of the United States of America. 2020;117(9):4971-82. Epub 2020/02/23. doi: 10.1073/pnas.1913904117. PubMed PMID: 32075919.
