# Supplementary material for: Interaction between coxsackievirus B3 infection and α-synuclein in models of Parkinson’s disease
Source: PLoS Pathog. 2021 Oct 25;17(10):e1010018. doi: 10.1371/journal.ppat.1010018 (PMC8568191; doi:10.1371/journal.ppat.1010018)
Supplement: Attachment — Submitted filename: Responses to reviewers.docx [file ppat.1010018.s012.docx]

**Response to Reviewer #3**

**Part III**

1. I suggest the authors provide a sentence or two discussing the role of pancreatic disease in the morbidity and mortality of the CVB3 infected mice (demonstrated by the pancreatic pathology and the body weight loss) and the possibility that a-syn expression in the pancreas leading to a significant increase in CVB3 replication in those tissues explains the higher morbidity and mortality in the a-syn OE transgenic mice.  In addition, the crystalline arrays in the dSH-SY5Y cells should be in discussions of Figure 2E in the text.

**[Response]** We have already answered it in the previous responses to reviewers. The detailed response is the following:

We added the explanation of the pancreatic tissue damage of CVB3 infected mice (L. 282-285). Given that transgenic α-syn was not expressed in the pancreas, we did not add the possibility suggested by the reviewer #2. Additionally, we also added the explanation of viral particles shown in Fig 2E (L. 162-163).

**Response to Reviewer #4**

**Part I**

1. One suggestion is to change the title to state in a “model of PD” or in a “mouse model of

PD”.

**[Response]** As suggested by the reviewer #4, we changed the title to “Interaction between coxsackievirus B3 infection and α-synuclein in models of Parkinson’s disease.”
